# Influence of age and education on the Rivermead Behavioral Memory Test (RBMT) among healthy elderly

**DOI:** 10.1590/S1980-57642016DN10100005

**Published:** 2016

**Authors:** Nicole Maineri Steibel, Maira Rozenfeld Olchik, Mônica Sanches Yassuda, Geisa Finger, Irênio Gomes

**Affiliations:** 1 Doutoranda do Programa de Gerontologia Biomédica da Pontifícia Universidade Católica do Rio Grande do Sul, Porto Alegre RS, Brasil; 2 Professora Adjunto do Curso de Fonoaudiologia da UFRGS, Departamento de Cirurgia e Ortopedia, Doutora em Educação e Mestre em Fonoaudiologia, Porto Alegre RS, Brasil; 3 Escola de Artes, Ciências e Humanidades, Universidade de São Paulo, São Paulo SP, Brasil; 4 Mestranda do Programa de Pós-Graduação em Gerontologia Biomédica - Instituto de Geriatra Gerontologia da Pontifícia Universidade Católica do Rio Grande do Sul, Porto Alegre RS, Brasil; 5 Programa de Pós-Graduação em Gerontologia Biomédica do Instituto de Geriatria e Gerontologia da Pontifícia Universidade Católica do Rio Grande do Sul, Porto Alegre RS, Brasil

**Keywords:** memory, aging, educational level, neuropsychological tests, memória, envelhecimento, escolaridade, testes neuropsicológicos

## Abstract

Memory is a cognitive domain extensively evaluated in the neuropsychiatric setting. Assessment tools with appropriate norms for age and educational level are necessary for the proper interpretation of results.

**Objective:**

To present normative data for older adults stratified by age and education for the Rivermead Behavioral Memory Test (RBMT). The effect of age and education on the total and sub-test scores was also analyzed.

**Methods:**

A cross-sectional study involving a sample of 233 healthy elderly from a third-age group in Porto Alegre with an average age of 70 (SD 7.9) years and 10.7 (SD 4.8) years of education was carried out. The RBMT is considered an ecologically valid memory test, since it includes tasks similar to everyday situations. The sample was stratified into the following age groups: 60-69 years, 70-79 years and > 80 years. The sample was also divided into individuals with < 8 years and ≥ 8 years of education. Pearson's Chi-squared test and Spearman correlations were used.

**Results:**

The elderly participants with low educational level had worse performance on all sub-tests, except the Pictures, Messages, Belongings and Orientation. Older elderly performed worse for total RBMT score and on the Face Recognition, Immediate and Delayed Route, Messages and Belongings subtests (p ≤ 0.005).

**Conclusion:**

Education and age significantly influenced RBMT scores. Therefore, norms for this test should be stratified according to these factors.

## RESUMO

A memória é um domínio cognitivo amplamente avaliado no contexto neuropsiquiátrico. Instrumentos de avaliação adequados com normas por idade e escolaridade são necessárias para interpretação correta dos resultados.

**Objetivo:**

Apresentar dados normativos para idosos estratificados por idade e escolaridade para o Teste de Comportamental de Memória Rivermead (RBMT). O efeito da idade e da escolaridade também foi analisado.

**Métodos:**

Estudo transversal com uma amostra de 233 idosos saudáveis, provenientes de um grupo de terceira idade do Município de Porto alegre, com média de idade de 70 anos (7,9 DP) e 10,7 (4,8 DP) anos de escolaridade. O RBMT é considerado um teste ecológico de memória, pois inclui tarefas semelhantes a situações cotidianas. A amostra foi dividida em idosos entre 60-69 anos, 70-79 anos e acima de 80 anos. A escolaridade foi dividida entre idosos abaixo de 8 anos e maior ou igual a 8 anos de estudo. Foram utilizados os testes Qui-Quadrado de Pearson e teste de correlação de Spearman.

**Resultados:**

Os idosos com baixa escolaridade apresentaram pior desempenho em todas as tarefas, exceto no reconhecimento de figuras, envelope, pertence e na orientação. Idosos mais velhos tiveram desempenho inferior no escore total do RBMT e nas tarefas de reconhecimento de faces, no caminho imediato e recente, no recado e pertence (p ≤ 0,005).

**Conclusão:**

A escolaridade e a idade influenciam significativamente nos escores do RBMT e as normas para este teste devem ser estratificadas de acordo com estes fatores.

## INTRODUCTION

The increase in longevity is being accompanied by diseases such as neurocognitive disorders.^1^^-^ 3 Despite advances in scientific research on cognitive aging, few adapted and validated instruments are available for Latin American countries. This creates a need for greater availability of valid, reliable, standardized and normalized instruments for use in the Brazilian elderly population.^4^^,^ 5 The interpretation of neuropsychological results should consider education, age, socioeconomic context and cultural background of the patient.^6^

Studies have shown that educational level has a significant impact on cognitive performance, which may confound the interpretation of test results, even those with ecological validity.^7^^-^ 9 In Brazil, there is significant heterogeneity in relation to cultural and socioeconomic status, making it especially important to develop, adapt and standardize cognitive tests that take into account at least educational level in the interpretation of their results.^7^^,^ 10 ^-^ 12

Memory is a cognitive domain widely investigated in the neurological and psychiatric setting. Thus, the adequacy of norms considering age and education is considered important for proper interpretation of results. The Rivermead Behavioural Memory Test (RBMT) is distinguished among several memory batteries for being presumably an ecologically valid memory test, as it replicates memory demands frequently faced in everyday life. It has been widely used for memory assessment in aging^11^^,^ 16 ^,^ 17 and can be very useful for the diagnosis of neurocognitive disorders alone and also as part of formal cognitive batteries. 

RBMT sub-tests resemble daily tasks involving visual and auditory episodic memory, such as memorizing a short story, faces and objects. Moreover, it is one of the few memory tests that evaluates prospective memory, which has been shown to be important in identifying early cognitive changes.^17^ Studies report that the RBMT has appropriate psychometric properties and high accuracy for distinguishing elderly people with preserved cognition from those with neurocognitive disorders.^11^^,^ 17 However, to date, no RBMT norms are available for Brazilian elderly.

Thus, the aim of this study was to suggest normative data for older adults for the RBMT, stratified by age and educational level. The effect of age and education on the RBMT total and sub-test scores was also investigated.

## METHODS

This is an observational cross-sectional study. Participants were drawn from companionship groups (public and private) for senior citizens in the city of Porto Alegre. This study was part of a larger study on the cognitive profile of community-dwelling older adults living in the city of Porto Alegre. Elderly people of different socioeconomic status and education were included in the sample. All volunteers who participated in the study signed the informed consent form. The study was approved by the Central Ethics Committee of the Federal University of Rio Grande do Sul under No. 23866

Inclusion criteria were: age over 60 years, normal cognitive profile. Individuals with a history of neurological disease, illiterate, or with language and/or hearing difficulties that prevented completion or understanding of the test instructions were excluded. The normal cognitive profile was determined by a multidisciplinary team based on the following criteria: absence of memory complaints, absence of impairment in instrumental activities of daily living, performance on the Mini-Mental State Examination above the education-adjusted cut-off scores, and not meeting DSM-V diagnostic criteria for dementia.

The RBMT was created by Baddeley, Cockburn and Wilson (1985). The test is divided into the 12 sub-tests Names, Belongings, Pictures, Story (immediate and delayed), Faces, Route (immediate and delayed), Messages (immediate and delayed), Orientation and Date. For each task, scores range from 0 to 2, where two points indicate normal performance; one point intermediate performance and zero points indicates error on the task. The RBMT profile score ranges from 0 to 24 points. The screening score (points 0-12) was not used in this study. The version employed was adapted to Brazilian Portuguese.^22^

The test was applied , individually, with an average duration of 30 minutes. The data collection period was from March 2010 to July 2014. The sample was divided into older adults aged 60-69 years, 70-79 years and 80 years or older. For educational level, the sample was divided into individuals with < 8 years' education and ≥ 8 years.

Statistical analysis was performed using the SPSS software (Statistical Package for Social Sciences) version 18.0. First, descriptive analyses of the RBMT variables were conducted, presenting mean and standard deviation or median and interquartile range for quantitative variables, and absolute and relative frequencies for categorical variables. The ANOVA and t-tests were used to compare the distribution of the total score by age group and level of education, respectively. Spearman correlations were used to evaluate the association between age and educational level and RBMT profile scores.

## RESULTS

The study included 233 healthy older adults, 28 men and 205 women, with mean age of 70.7 ± 7.9 years and mean education of 10.75 ± 4.8 years. Individuals with educational level below 8 years corresponded to 28.8%, and with over eight years, 71.2%. Sociodemographic characteristics are given in Table 1.

**Table 1.** RBMT scores according to sociodemographic characteristics.

**Demographic data**		**n (%)**	**RBMTM ± SD**	**p value**
Age group	< 69 years	113 (48.5)	19.0 ± 3.8^a^	< 0.001*
	70-79 years	89 (38.2)	17.2 ± 3.9^b^
	> 80 years	31 (13.3)	15.6 ± 4.0^b^
Education	< 8 years	67 (28.8)	15.6 ± 4.	<0.001**
	≥ 8 years	166 (71.2)	18.0 ± 3.6	
Sex	Men	28 (12)	17.4 ± 3.9	0.539**
	Women	205 (88)	17.9 ± 4.1	
MMSE		27.6 (± 2.4) 18-30		
Total		233 (100)	17 ± 4.1	

*ANOVA. ** t-test. ^a^ANOVA: This group is statistically different from the oldest group. ^b^ANOVA: These groups are statistically similar.

The analyses showed significant differences between educational levels and age groups, with worse performance for the group with low education. Regarding age, individuals younger than 69 years of age had lower scores than the other age groups.

Correlational analyses showed negative associations with most RBMT sub-tests and total score (except Messages, Date and Orientation) and positive associations with the total and most sub-test scores (except Pictures and Belongings) (Table 2).

**Table 2.** Correlation of tasks with age and educational level

**Tests**	**Age**		**Educational level**	
	ρ	p*	ρ	p*
1. Immediate Story	-0.175	0.007	0.266	0.000
2. Delayed Story	-0.176	0.007	0.300	0.000
3. Pictures	-0.143	0.029	0.074	0.265
4. Faces	-0.211	0.001	0.234	0.000
5. Immediate Route	-0.153	0.019	0.215	0.001
6. Delayed Route	-0.214	0.001	0.228	0.000
7. Immediate Messages	-0.030	0.653	0.126	0.056
8. Names	-0.150	0.022	0.188	0.004
9. Delayed Messages	-0.196	0.003	0.264	0.000
10. Belongings	-0.236	0.000	0.084	0.201
11. Date	-0,092	0.160	0.248	0.000
12. Orientation	-0.020	0.761	0.123	0.061
Total	-0.329	0.000	0.240	0.000

*Spearman's correlation coefficient.

Table 3 shows the distribution of values for total RBMT score stratified by age and level of education. Medians for RBMT scores and groups with < 8 years' education and ≥ 8. Years are given. In an initial analysis, no significant differences were found between the age groups 70-79 years and > 80 years, therefore these two groups were analyzed together, as shown in Table 3.

**Table 3.** Distribution of RBMT values according to age group and schooling in 233 elderly without cognitive decline.

**Group**		**N (%)**	**Range (min-max)**	**Median(± IQ)**	**M ± SD**
Age 60-69 years	< 8 years' education	21 (9.0%)	4-24	17.6 (12.5-20)	16.1 ± 4.7
	≥ 8 years' education	92 (39.5%)	6-24	20.2 (17.25-22)	19.6 ± 3.3
Age ≥ 70 years	< 8 years' education	46 (19.7%)	7-22	15.0 (12-18)	15.0 ± 3.7
	≥ 8 years' education	74 (31.8%)	9-24	18.7 (14.75)	17.9 ± 3.8

## DISCUSSION

The results showed that both age and level of education had a significant impact on RBMT performance. Results suggested the existence of a negative correlation between age and performance on the RBMT and a positive correlation with education. Most studies using the RBMT have been conducted in groups of elderly with Alzheimer's disease and mild cognitive impairment.^12^^,^ 16 ^,^ 17 In studies published since 1998, the RBMT was shown to be a memory test capable of differentiating these clinical groups from healthy controls, these studies.^24^ Some translations were found for the RBMT into Japanese but different scoring values are ​​found in the Brazilian population.^23^ Thus far, we have found no studies comparing the RBMT with the MMSE, only with other mostly verbal tests. However, all the articles showed that the RBMT is a good test for discriminating different clinical groups. A study in a Turkish population found the RBMT to be reliable, valid and suitable for use in patients with acquired brain injury. The authors also provided a valid unidimensional summed score.^25^ In Brazil, a previous study involving the RBMT showed a modest effect of education on healthy subjects' performance on the test.^10^ The results showed no differences on the RBMT among healthy controls with different levels of education. It is believed that these findings may be due to the small sample in each group (n = 22 for aged individuals with less than eight years of education and n = 23 for up to nine years). In the present study, scores for the population of the same educational level were 67 and 166, respectively. One of the few studies in the literature which also used the RBMT in a sample of healthy individuals showed a significant correlation with education.^18^ However, the correlation found was greater for other memory tests used (Wechsler Memory Scale, and Everyday Memory Questionnaire) than for the RBMT. Also, other studies conducted in Brazil have indicated that the RBMT is less influenced by educational experience than other cognitive tests such as the CAMCOG, the MMSE and the Wechsler Intelligence Scale Revised-WAIS-R.^11^

The MMSE was applied only as a criterion for inclusion by ruling out dementia. However, it was evident that in our population the mean neared the ceiling effect, and the RBMT appeared to be more sensitive for some tasks.^23^^,^ 24 The values of the MMSE were adjusted for age and education in accordance with Brazilian references.^26^ It is important to highlight that some RBMT sub-tests (Faces, Pictures and Name) were not influenced by education levels in a previous study.^18^ Similarly, in the present study, the Pictures and Orientation sub-tests were not influenced by educational level.

According to other studies, low educational level affects cognitive skills, as well as complex activities of daily living.^19^ Several studies have shown an association between education and cognitive performance. Our findings are not in agreement with previous studies where the effects of educational level was non-linear, tending to plateau, as marked increases in performance are not expected beyond 10 years of study.^21^ The findings of this study are relevant, showing that even ecological cognitive batteries may be influenced by education and age in developing countries such as Brazil.

Futures studies should investigate the RBMT profiles in a clinical sample and provide cut-off scores, aspects which could not be incorporated in the present study.

In conclusion, normative data were suggested for age and educational levels. Results suggest these aspects need to be taken into consideration in the context of the neuropsychological evaluation of Brazilian elderly.
